# Helping Postpartum Women in Mali Achieve Their Fertility Intentions: Perspectives From Introduction of the Dedicated Postpartum IUD Inserter

**DOI:** 10.9745/GHSP-D-18-00138

**Published:** 2018-10-03

**Authors:** Eva Burke, Marie Léa Dakouo, Laura Glish, Pierre Moon, Paul D. Blumenthal

**Affiliations:** a Independent consultant, Bath, England.; b Population Services International Mali, Bamako, Mali.; c Population Services International, Washington, DC, USA.; d Population Services International, Washington, DC, USA, and Stanford Program for International Reproductive Education and Services (SPIRES), Department of Obstetrics and Gynecology, Stanford University School of Medicine, Palo Alto, CA, USA.

## Abstract

**Background:**

Mali has one of the highest maternal mortality ratios in the world coupled with one of the lowest modern contraceptive use rates. Nearly a quarter of the country's 750,000 annual births occur within 24 months of a previous birth, increasing the risks for mothers and babies. Nearly 70% of postpartum women have an unmet need for family planning. In 2016, Population Services International Mali (PSI-Mali) introduced a dedicated postpartum intrauterine device (PPIUD) inserter to replace the technique of using forceps for PPIUD insertion, with the aim of helping to address this substantial family planning gap.

**Methods:**

A mixed-methods approach was used to assess program results and the experiences of PSI-trained providers using the dedicated PPIUD inserter in 5 health facilities in Bamako. We conducted 10 key informant interviews with providers and 4 key informant interviews with operational and clinical staff involved in training and supporting providers. Further data were collected from district health surveys and facility registers. Secondary data encompassed documentation from 2011 through 2017, with the service delivery figures of PPIUD using the dedicated inserter focused on the pilot period of March 2016 through December 2017. Primary data were collected in Mali in July 2017.

**Results:**

Between March 2016 and December 2017, PSI-Mali trained 134 providers on the dedicated PPIUD inserter and provided more than 3,500 voluntary PPIUDs. Of the 1,840 voluntary PPIUDs provided in 2017 alone, 67% were provided by facilities trained to use the dedicated PPIUD inserter. Providers stated a preference for the inserter (compared with the use of forceps) due to its ease, speed, and perceived lower associated risks of infection. Service data from the 5 facilities visited showed an overall average PPIUD uptake of 7.3% of deliveries in 2017. Although private facilities had considerably fewer deliveries than public facilities (600–900 compared with 20–30, respectively), a much higher proportion of women delivering in the private facilities chose a PPIUD.

**Conclusion:**

The acceptance of the dedicated PPIUD inserter by providers may help reduce some of the supply-side barriers that inhibit women's access to postpartum family planning methods. With continued support to providers, coupled with ongoing efforts to address differences in service trends between sectors and demand-side barriers to the PPIUD and family planning more broadly, the dedicated PPIUD inserter could play an important role in responding to the high unmet need among postpartum women in Mali.

## Abstract (precis)

In this pilot introduction setting, trained providers reported higher acceptance and preference for the dedicated inserter compared with the conventional postpartum insertion with forceps, suggesting potential for the dedicated inserter to expand access to postpartum IUDs.

Résumé en français à la fin de l'article.

## INTRODUCTION

Birth intervals of less than 24 months are associated with increased risk of maternal, infant, and child mortality.^1^^,^^2^ Conservative estimates suggest that 9% of the 11 million annual deaths among children under 5 years of age could be averted if birth intervals were 24 or more months apart.^3^ An estimated 214 million women worldwide have an unmet need for family planning,^4^ with women in the extended postpartum period—defined by the World Health Organization (WHO) as the first 12 months following childbirth^5^—among those with the greatest need. Family Planning 2020 (FP2020) estimates that over 90% of postpartum women in developing countries want to space or limit a subsequent pregnancy, but 61% do not use a family planning method.^6^

Mali has among the highest maternal mortality ratios and infant mortality rates in the world: 587 per 100,000 live births^7^ and 75 per 1,000 live births,^8^ respectively. The fertility rate remains high at 6.1 children per woman and the modern contraceptive prevalence rate is one of the world's lowest at 14%.^9^ More than 1 in 4 women have an unmet need for family planning: 19% for spacing and 7% for limiting births.^10^ Analysis of Demographic and Health Survey (DHS) data from 2006 revealed that nearly 70% of postpartum women in Mali have an unmet need for family planning.^11^ Because postpartum abstinence is practiced for a relatively short period of time (median 2.2 months), half of women are at risk of becoming pregnant again 11.7 months after giving birth.^10^ Nearly a quarter of the annual 750,000 births in Mali occur within less than 24 months after the last birth,^10^ representing a missed opportunity to significantly reduce maternal and child mortality by lengthening birth intervals.^3^ About 48% of pregnant women attend antenatal care (ANC) visits at least once, with 38% attending at least 4 times.^12^ Although, nearly 60% of births are attended by a skilled birth attendant,^13^ only 40% of women and neonates receive postnatal care in the 2 days following delivery.^10^ These patterns of contact between women and their ANC providers suggest strong potential for the increased role of voluntary postpartum family planning (PPFP) in helping women meet their fertility intentions within the context of access to a range of methods that are medically appropriate for immediate postpartum women.

Access to information and counseling during ANC visits could increase the likelihood of PPFP uptake immediately following childbirth.^5^ According to WHO *Medical Eligibility Criteria for Contraceptive Use*,^14^ the copper intrauterine device (IUD) is one of the immediate PPFP options for breastfeeding women. The postpartum IUD (PPIUD) is a highly effective, low-cost contraceptive option—lasting up to 12 years but reversible at any time—for women in the immediate and extended postpartum period. The PPIUD can be inserted within 10 minutes of delivery of the placenta before a woman leaves the delivery room (postplacental), during a cesarean delivery (intracesarean), or up to 48 hours (immediate) postpartum.^15^ Immediate insertion of the IUD postpartum may be beneficial for women as it allows them to access the method prior to leaving the health facility, making some of the side effects typically associated with the IUD less noticeable.^15^

In 2011, Population Services International Mali (PSI-Mali) launched voluntary PPIUD services using conventional IUDs (the Copper T380A) packaged for interval insertions in health facilities with a high volume of deliveries. Between 2011 and 2015, 141 health providers and stakeholders—21 from the private sector and 121 from the public sector—from Bamako, Kayes, and Sikasso participated in PSI-Mali's PPIUD training program. During this initial phase of the program, more than 2,300 women volunteered to be provided with PPIUDs; however, the use of conventional IUDs for immediate postpartum women came with certain challenges. In low-income countries, access to commonly recommended forceps for PPIUD insertions can be limited, as they are not usually found in the maternity ward. Additionally, sterilizing the forceps can contribute to an increased workload and cost for providers in busy obstetrical units and reduce the likelihood of provision of PPIUDs to women. The insertion of conventional PPIUDs with forceps involves extra manipulation of the IUD, which can increase risk of contamination, possible subsequent infection, and/or the potential for damage to the IUD.^16^

Previous studies reveal that although PPIUDs are highly effective, their expulsion rates are typically higher than with interval IUDs.^17^ One reason for this is that, due to the shape of the postpartum uterus, the strings from the conventional IUDs are too short to be visible once the PPIUD is inserted, thereby inhibiting the provider's ability to know whether the PPIUD is correctly placed. To address this challenge, PSI collaborated with the Stanford Program for International Reproductive Education and Services (SPIRES) and Pregna International Ltd to develop a new dedicated PPIUD inserter. The new product eliminates the need for forceps by elongating the insertion tube, which is firm but bends to accommodate the shape of the postpartum uterus and has a longer string that is visible after PPIUD insertion.^18^ The IUD product itself remains the same (the Copper T380A), with only the insertion packaging differing. Initial trials in India found that the dedicated PPIUD inserter was effective and safe and had high acceptability among women and providers.^16^ However, the trials did not allow for a comparative analysis of provider or client experiences or an exploration of their preferences between the different insertion techniques, which might have influenced the provision and uptake of the PPIUD. Results of a recent randomized controlled trial in India comparing experiences with the dedicated PPIUD inserter and forceps indicated high provider and patient acceptability and comparable complete expulsion rates.^19^

The new PPIUD inserter eliminates the need for forceps by elongating the insertion tube and having a longer string that is visible after insertion.

PSI-Mali's pilot program was the first in sub-Saharan Africa to introduce the dedicated PPIUD inserter, with the aim of improving delivery of voluntary PPIUD services. The pilot set out to generate lessons learned to improve programming, inform a national strategy for increasing availability of voluntary PPIUDs in Mali, and provide a valuable road map for the introduction and scale up of PPIUD access in other country contexts. A year after the launch of the dedicated PPIUD inserter in Mali, PSI commissioned this case study to analyze internal program results and their wider implications for increasing access to voluntary PPIUD service delivery in other PSI programs. The Mali pilot project provides a unique opportunity for a comparison of a voluntary PPIUD service delivery program using the conventional IUD inserted with forceps with a service delivery program using the dedicated PPIUD inserter. This case study shares lessons from the pilot that can be applied broadly to PPIUD acceptability and uptake, with a view to increasing the method mix for postpartum women wishing to space or limit future pregnancies.

## METHODS

We used a mixed-methods approach to assess program results to date as well as the experiences of PSI-trained providers using the dedicated PPIUD inserter. The data collected included a review of PSI documentation and secondary data from the PSI management information system, District Health Information System 2 (DHIS 2), and facility-level registers in PSI-trained facilities as well as primary data collected through key informant interviews. The secondary data review examined documentation produced between 2011 and 2017, which allowed a review of trends before and after the introduction of the dedicated PPIUD inserter.

Key informant interviews were conducted to collect provider perspectives on the dedicated PPIUD inserter and to explore the perceived drivers behind PPIUD uptake. Purposive sampling was used to identify public and private facilities and providers based on a range of criteria, specifically that providers have been trained in PPIUD insertion with forceps and with the dedicated inserter by PSI-Mali; facilities have a relatively high volume of deliveries; and providers work in a facility where PPIUD service provision is high, medium, or low to assess differences and outliers. The total sample was 10 providers—6 midwives, 2 gynecologists, and 2 doctors—trained on the dedicated PPIUD inserter who were based in 5 different health facilities in Bamako: 3 public referral health centers, known as *Centres de Santé de Reference*, and 2 private sector Protection de la Famille (PROFAM)-branded clinics from PSI-Mali's social franchise network. The names of facilities and providers have been omitted from this article to respect the confidentiality and anonymity of the interview informants. Instead, the facility names have been replaced with code names: Public A, B, and C for the 3 public health facilities and Private A and B for the 2 PROFAM clinics. To complement provider perspectives of the dedicated PPIUD inserter, 4 PSI-Mali operational and clinical staff who worked directly on the PPIUD program were also interviewed. The primary data that inform this case study were collected in Mali in July 2017, and the secondary data were later updated with the most recent PSI data from DHIS 2. After the interviews were transcribed, manual thematic analysis of the transcripts was conducted and codes entered into a data analysis framework to highlight key themes.

District- and facility-level data and provider interviews were analyzed to better understand PPIUD trends and experiences.

### Ethical Considerations

Because the purpose of this case study was determined to be for internal programmatic improvement, it did not meet the definition of human subjects research needing the review of the Institutional Review Board. However, key steps were taken prior to meeting with the providers PSI-Mali had trained on the dedicated PPIUD inserter to ensure data collection was conducted in an ethical manner. Health authorities in the Ministry of Health (MOH) and the participating facilities were informed of the objectives of the case study and their approval was sought prior to conducting interviews with providers. The research team explained to all potential interview participants the objectives of the interview, any potential risks and benefits, that any information provided would be confidential and anonymous, and that their participation was voluntary. All providers provided written informed consent prior to participating in an interview. The MOH provided written support to publish the study results.

## INTRODUCTION OF THE DEDICATED PPIUD INSERTER

In March 2016, with 5 years of experience supporting the introduction of voluntary PPIUD (using a forceps insertion technique) into the package of maternal health services, PSI-Mali gained support from the MOH to introduce the dedicated PPIUD inserter in a pilot study. The study allocated 2 midwives to support the pilot phase, including rolling out the training program and providing follow-on supportive supervision. With the assistance of MOH stakeholders, sites were chosen to pilot the dedicated PPIUD inserter. A select group of 18 providers from 10 public health facilities were initially trained. The facilities were chosen based on meeting the following key criteria:

- A sufficiently high volume of deliveries to increase the likelihood of higher volume of voluntary PPIUD services, enabling providers to build and maintain their skills
- Experience in PPIUD insertions with forceps, or interval IUD insertions as a minimum
- Demonstrated commitment from providers to counsel and provide voluntary PPIUD

While the trainings largely focused on clinical aspects of the new insertion technique, as providers were already experienced in the provision of PPIUD (with forceps), refresher trainings were also provided within the context of high-quality family planning counseling on the range of methods suitable for postpartum women.

Prior to the provider trainings, an orientation was conducted at each pilot site to allow a range of members of staff—from center managers to midwives and nurses—to learn about the dedicated PPIUD inserter. These sessions helped to ensure the staff had the necessary information to inform women during visits to a health facility—for vaccinations, family planning counseling, or ANC—to raise general awareness. Site orientation was done in collaboration with the MOH's 2 allocated public-sector midwives. As part of the initial introduction, and to generate evidence of the efficacy of the PPIUD with the dedicated inserter over a 4-month period, PSI-Mali monitored clinical data related to insertions, including the timing of the insertions and any postinsertion complications or side effects. Between March and June 2016, PSI-Mali identified 10 cases of PPIUD expulsions out of 343 insertions (2.9%), lower than the proportion found in the original pilot study in India (7.5%)^18^ and in previous studies of PPIUD insertions with forceps, which ranged from 6.9% to 8%.^20^^–^^22^ Possible explanations for the difference in expulsion rates observed in this and the Indian studies include: (1) in the Indian studies, resident physicians had less experience in PPIUD insertion than participating providers in Mali, and (2) as a result of the early Indian experience, adjustments were made to the insertion technique in Mali, particularly trimming the strings as close as possible to, or just inside, the cervical os.^19^ Training sessions conducted during this initial introductory phase also allowed PSI-Mali to identify experienced and motivated providers to be trained as trainers, thus growing the pool of trainers for the successive rollout and supervision of the dedicated PPIUD inserter program. Between March 2016 and December 2017, PSI-Mali trained 134 providers on the dedicated PPIUD inserter across 4 regions (Table).

**TABLE.** Public and Private Providers Trained in the Dedicated PPIUD Inserter in Mali, by Region

Region	No. of Public Providers	No. of Private Providers
Bamako	42	49
Kayes	6	14
Sikasso	12	3
Mopti	8	0
**Total**	**68**	**66**

PSI used provider training and staff orientation sessions to raise awareness and ensure that female patients would receive the correct information about PPIUDs.

Together, PSI-Mali and the MOH conducted follow-on supportive supervision visits in the weeks after training as well as at the midpoint and in the final weeks of the pilot period. Public and private facilities received the same supportive supervision schedule and content. This included:

- **Community sensitization:** support conducting community sensitization events; information, education, and communication materials; training MOH community health workers
- **Supportive supervision:** all trained providers receive post-training supervision and ongoing supportive supervision in clinical and counseling skills from PSI-Mali supervisors and/or the pool of trained providers
- **Supplies:** the dedicated PPIUD inserter and all the necessary commodities for insertion were provided free of charge to facilities by PSI-Mali
- **Record keeping and data collections support:** support on data reporting and monthly collection of PPIUD service data

## RESULTS

Results presented are from March 2016 through December 2017, unless otherwise stated, and are based on PSI service data in DHIS 2 and/or facility-level registers.

### Provider Preferences for the Dedicated PPIUD Inserter

All providers interviewed stated a preference for the dedicated PPIUD inserter compared with inserting PPIUD with forceps for the following reasons:

- PPIUDs were easier and faster to insert
- The inserter was more convenient
- The procedure required fewer materials and no sterilization
- Patients were at less risk of infection or perforation
- Providers were confident that the PPIUD was placed correctly due to the visibility of the strings
- The procedure was perceived as less painful for women

Providers preferred the dedicated PPIUD inserter because it was more convenient, faster, and required fewer materials.

While no provider relayed any major difficulties with inserting the PPIUD using forceps, providers expressed a much more favorable opinion of the dedicated inserter. Respondents commonly cited ease of use and reduced perceived associated risks with the new product, giving them more confidence to provide the voluntary PPIUD service:

*[Providers] accept this method because of its ease of use. We know this influences service delivery. First, people must accept it … when staff are skeptical of a method, it is very hard to make it work. [The staff] have much more confidence with the new inserter*. (Gynecologist, public-sector facility, Bamako)

The rarity of complications or adverse reactions related to the dedicated PPIUD inserter also contributed to greater provider confidence. While some cases of discontinuation were reported, these were largely due to husbands wanting the IUD removed.

Several providers explained that, in their experience, if women see or hear the forceps, they often get nervous. Providers relayed instances of women changing their mind about wanting the PPIUD once they saw the forceps or nervously moving while the forceps insertion was taking place, raising provider fears of causing a perforation:

*[The new inserter] is easier. It is less stressful for the woman, and for you … if the woman sees the forceps, she is scared. She doesn't stay still … we are scared to perforate her when she doesn't sit still*. (Midwife, public-sector facility, Bamako)

### Uptake of Voluntary PPIUD Services

**FIGURE 1:**
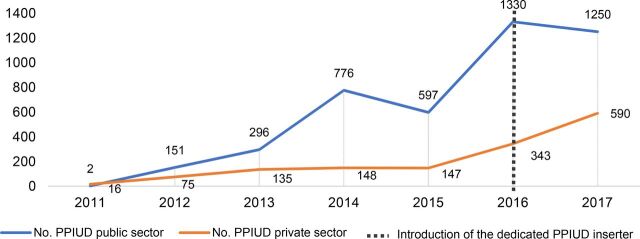
Program-Wide Voluntary PPIUD Services Provided November 2011 Through December 2017 Abbreviation: PPIUD, postpartum intrauterine device; PSI, Population Services International.

Voluntary provision of PPIUD services in the public and private sectors has steadily increased since PSI-Mali launched the first PPIUD program in late 2011. Uptake of PPIUD services saw a substantial increase in 2016 when PSI-Mali introduced the dedicated inserter, with providers delivering 1,673 voluntary PPIUD services compared with 744 in 2015. In 2017, 1,840 voluntary PPIUD services were provided, surpassing the 2016 results. In total, 3,513 voluntary PPIUD services were provided between 2016 and 2017 over the course of the pilot period. Figure 1 illustrates the trends in voluntary PPIUD uptake from the start of the voluntary PPIUD program launch in 2011 through December 2017.

While services provided in the public sector have increased over time, uptake trends have been variable, rising and falling throughout over the last 4 years. In 2017, however, the public sector nearly sustained levels of uptake compared with 2016, and substantially surpassed the uptake recorded in 2015, the year before the dedicated inserter was introduced. Prior to that, peak results for the public sector were in 2014 and 2016, which coincide with a substantial number of trainings conducted for public-sector staff (41 providers trained in 2014 and 48 in 2016). In the trainings, each provider was required to counsel and provide 3 to 5 voluntary PPIUD insertions under supervision to qualify as a certified provider. For the private sector, in 2017, uptake exceeded the number provided in 2016 and was substantially higher than in 2015. This can be attributed, in part, to the increased number of private-sector social-franchise facilities trained in the dedicated PPIUD inserter compared with the number of franchised facilities trained in the forceps insertion technique.

While Figure 1 provides program-wide results for voluntary PPIUD services provided with either forceps or the dedicated inserter, a snapshot of the data in 2017 reveals that out of the 1,840 voluntary PPIUDs provided, the majority (67%) were provided by facilities trained in voluntary PPIUDs with the dedicated inserter.

Of the 1,840 voluntary PPIUDs provided in 2017, 67% were provided in facilities trained to use the dedicated inserter.

**FIGURE 2:**
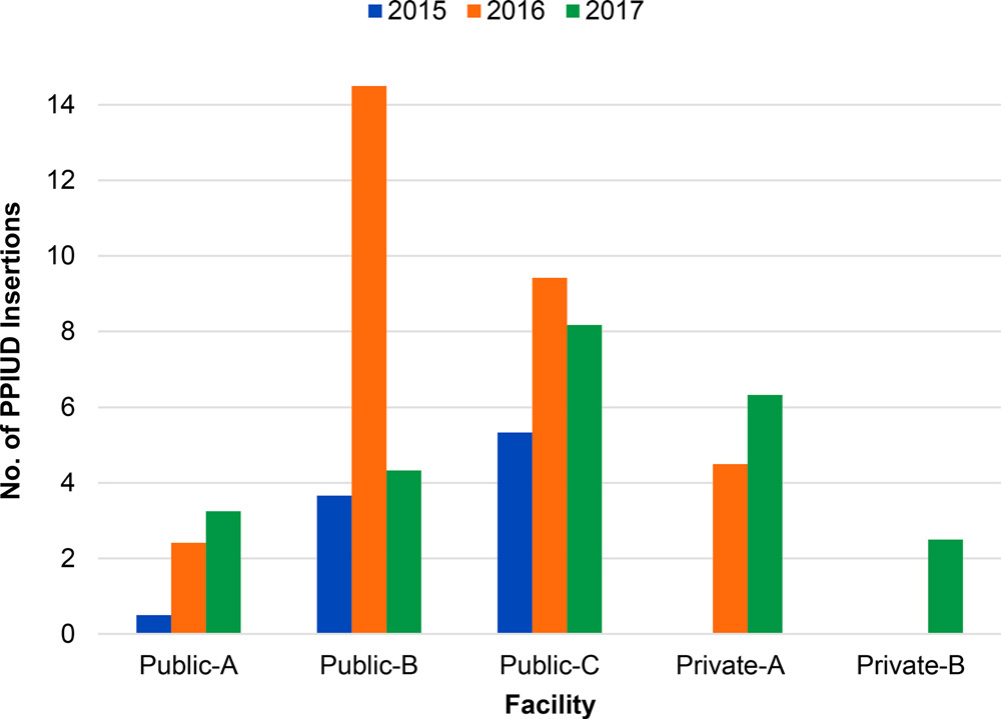
Average Number of Voluntary PPIUD Insertions per Month per Facility Among 5 Case Study Facilities, January 2015 Through December 2017 Abbreviation: PPIUD, postpartum intrauterine device.

Service data from the 5 facilities visited for the case study were analyzed, and results among the 3 public-sector facilities showed a peak in voluntary PPIUD service uptake in 2016, compared with 2015 and 2017, for 2 of the public facilities; the other showing an increase in services in 2017. In the 2 private facilities, no data were available for 2015, as either the provider had not been trained to provide voluntary PPIUD services or PPIUD data were not disaggregated from interval IUDs in client registers. However, since late 2016, the private facilities have showed promising trends in voluntary uptake. We calculated the average number of PPIUD insertions per month per facility to provide a comparative view of voluntary PPIUD uptake among the 5 case study facilities (Figure 2).

On average, the 5 facilities delivered 4.9 voluntary PPIUD services per month in 2017, compared with an average of 7.7 per month in 2016. Public facilities B and C provided the highest number of voluntary PPIUD services per month, especially in 2016. This was largely due to the practical training sessions taking place in their facilities, where numerous visiting providers had to each do 3 insertions to qualify as a trained provider in the dedicated inserter. The average number of PPIUD insertions per month in private facility A increased substantially in 2017 compared to 2016. Because private facility B was trained in late 2016, only 2017 service data were available.

### Uptake of Voluntary PPIUD Services as Proportion of Total Deliveries

**FIGURE 3:**
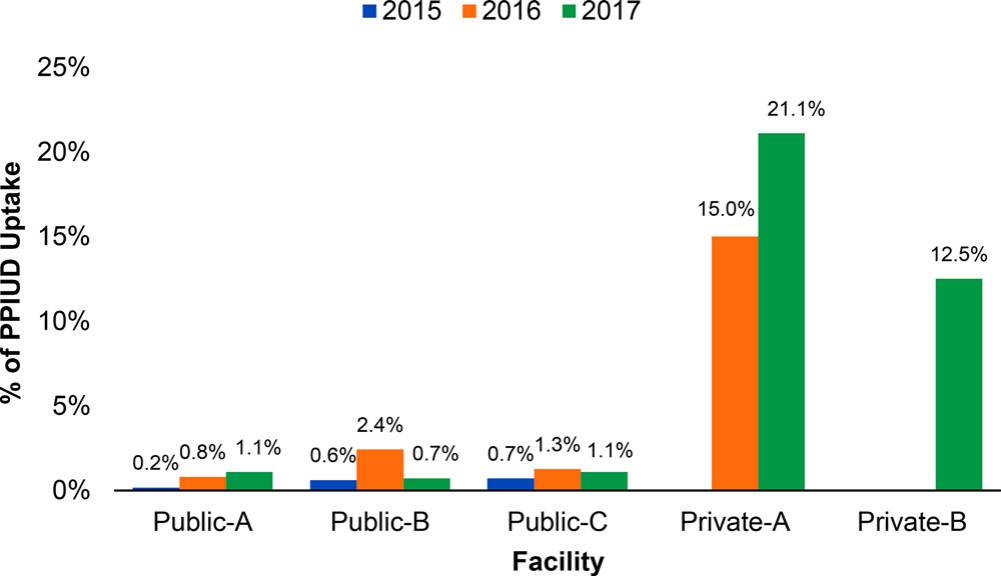
Voluntary PPIUD Uptake vs. Estimated Total Number of Deliveries Among 5 Case Study Facilities Abbreviation: PPIUD, postpartum intrauterine device.

Each of the 5 facilities included in the case study was asked to provide an estimated monthly average of deliveries at their facility based on tallies from their institutional log books. The public facilities estimated significantly more deliveries per month, between 600 and 900 deliveries, than did the private-sector facilities, which estimated 20 to 30 deliveries. Service data from the 5 health facilities showed an overall average voluntary PPIUD uptake of 7.3% for all deliveries in 2017 compared with 4.9% for 2016. This, however, masks variations between sectors. Voluntary PPIUD uptake as a proportion of total deliveries was considerably higher in the 2 private-sector facilities than the 3 public-sector facilities. Although private facilities have significantly fewer deliveries, they also have a much higher rate of uptake of voluntary PPIUD, defined as the number of services as a percentage of total women delivering at their facility (Figure 3).

Service data from the pilot facilities showed an overall average voluntary PPIUD uptake of 7.3% for all deliveries in 2017 compared to 4.9% for 2016.

PSI-Mali staff posit that, in the private sector, repeat interactions with the same provider for ANC visits and for delivery contributed to increased opportunity and/or increased trust between clients and providers that positively impacts discussion of PPFP. In the public sector, a woman may see several providers from different teams in the maternity unit throughout her pregnancy, delivery, and postpartum period.

### Volume of Services Provided by Number of Active Providers

To understand whether the introduction of the dedicated PPIUD inserter had an impact on the number of voluntary PPIUD services, it is also necessary to understand whether the number of providers offering the service was the same or different over time. This will shed light on whether observed increases in service uptake were due to having more providers trained to offer the service or higher PPIUD service volumes per provider.

**FIGURE 4:**
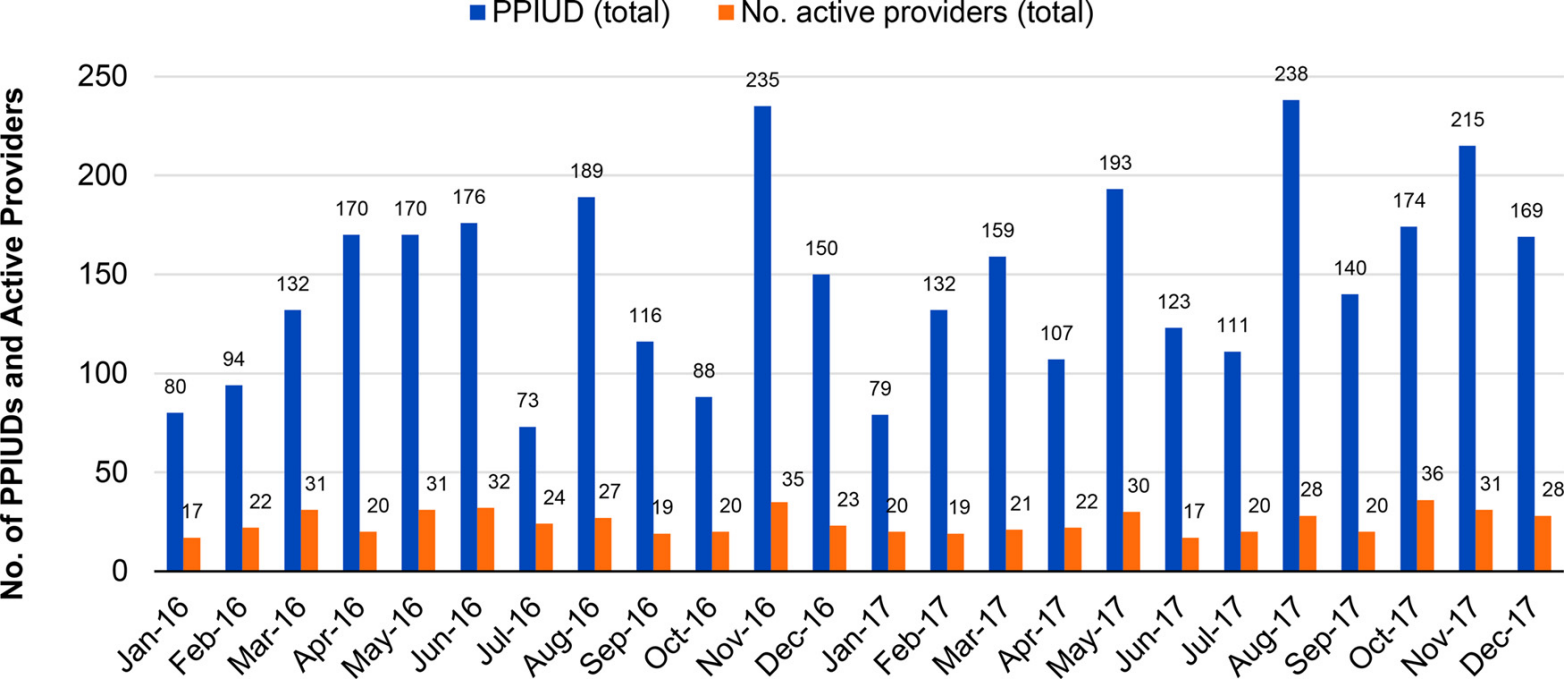
Number of Voluntary PPIUDs Provided and Number of Active Providers Abbreviation: PPIUD, postpartum intrauterine device.

**FIGURE 5:**
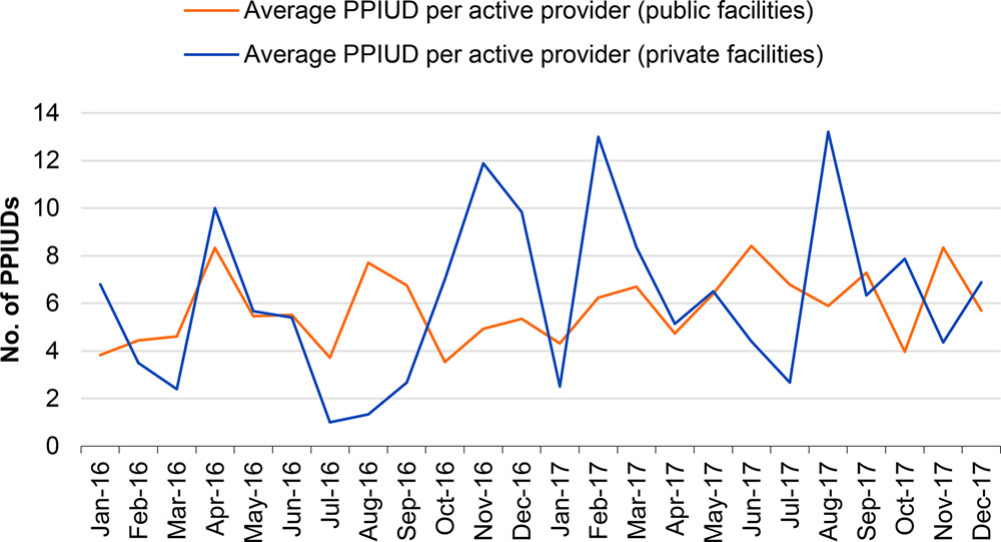
Average Monthly Voluntary PPIUDs per Active Provider, by Public and Private Facilities Abbreviation: PPIUD, postpartum intrauterine device.

However, in order to gain a snapshot of the pilot program as a whole, data on the total number of “active” providers—defined as providers delivering at least 1 voluntary PPIUD insertion in a given month—were compared with the total number of voluntary PPIUD insertions provided (Figure 4). This provided an estimate of the average number of PPIUD insertions per active provider by month for 2016 and 2017 (Figure 5).

Figure 4 shows that the number of active providers per month fluctuates between 17 and 36. The total number of voluntary PPIUD services provided varies more dramatically, ranging from 73 to 238. Broadly, most months with high uptake of PPIUD services coincided with a greater number (>30) of active providers. There were, however, some months—for example, April and August 2016 and March and August 2017—in which a lower number of active providers provided a higher number of voluntary PPIUD services, indicating higher PPIUD client volumes per provider. The data, therefore, do not provide a clear link between an increased number of providers trained in PPIUD and an increased number of voluntary PPIUD services provided.

The data did illustrate monthly fluctuations in the average number of voluntary PPIUD services provided per active provider by sector (Figure 5). More specifically, they showed a slightly higher average number of PPIUD services delivered per active provider in the private sector (6.2) compared with the public sector (5.8), and a much wider range of providers inserting voluntary PPIUDs: from 4 to 8 voluntary PPIUDs per provider per month in the public sector to between 1 to 13 PPIUDs provided in the private sector (Figure 5).

Compared to the public sector, the private sector had a higher average number of PPIUD services delivered per active provider and a much wider range of providers inserting voluntary PPIUDs.

Although the number of providers trained by PSI-Mali has increased over time, the data show that the number of providers actively providing voluntary PPIUDs each month fluctuates. In some months, trained providers provide no PPIUD services at all, despite no reported stock-outs during the pilot period. This finding was also observed during site visits. The most recent analysis of DHS data on birth seasonality in Mali shows births highest in April and June and lowest in January.^22^ While January 2016 and 2017 had the lowest total number of PPIUD insertions, overall patterns of insertions and births are not correlated, suggesting that birth seasonality might be a contributing factor but not a strong driver of the monthly insertion numbers.

Calculating the average monthly number of active providers who inserted PPIUDs in 2016 and 2017, reveals that, on average, 18.8 private providers and 5.9 public providers actively provided voluntary PPIUD insertions, regardless which approach was used. This represents only a small proportion of providers who have been trained in PPIUD insertions. Provider-level analysis also reveals that the same providers can fluctuate between being “active” and “inactive,” with few consistently providing voluntary PPIUDs each and every month.

### Provider Perceptions of Service Trends

Although not specifically related to the dedicated inserter, but to PPIUD insertions more generally (including with forceps), providers identified supply- and demand-side drivers and barriers associated with PPIUD use or non-use. Based on their experiences of providing PPIUD, their main perceptions were:

- **Wider site orientation increases access to PPIUDs:** Increasing the number of staff at a facility who can counsel women during ANC visits and delivery, or assist with voluntary PPIUD insertions, can contribute to increased access to PPIUDs.
- **ANC visits are an essential time to counsel women:** Providers reported that counseling women during ANC sessions was more likely to result in voluntary postplacental PPIUD insertions; however, if counseling was done in early labor or postpartum, women were less likely to choose this method.
- **Community sensitization increases acceptance of the IUD:** Providers believed that community sensitization caused a shift in the acceptance of the IUD, in general, as a method of family planning and had an impact on the uptake of voluntary PPIUDs.
- **Provider availability influences PPIUD service availability:** Providers reported that in public facilities, the high volume of deliveries, the quick turnover of new mothers in the delivery suites, and the relatively low number of providers trained in PPIUD insertions to date, meant that access to PPIUD is often restricted.
- **Retention and relocation of public-sector providers creates challenges for PPIUD availability:** Providers believed that public-sector staff or provider rotation, retirement, or sick leave can all negatively influence the continuous availability and provision of voluntary PPIUD services.
- **Demand-side barriers hinder uptake of voluntary PPIUD:** Providers reported the most common reasons for non-use of the PPIUD were husband opposition to family planning, women changing their mind post-delivery—having selected a PPIUD during ANC visits—because of fear of pain or wanting to discuss it with their husbands first, women preferring to wait 40 days to resume sexual activity and consider all family planning methods, and myths and negative perceptions about IUDs.

### Demographic Characteristics of PPIUD Users

**FIGURE 6:**
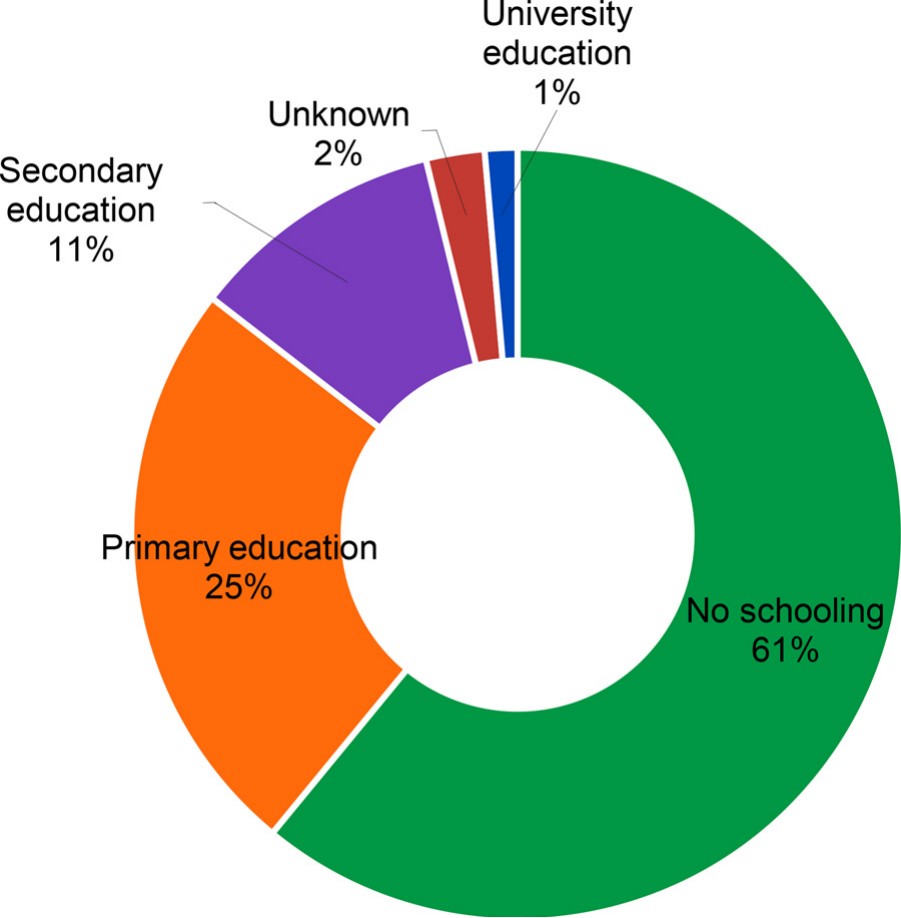
Education Status of Voluntary PPIUD Clients, 2017 Abbreviation: PPIUD, postpartum intrauterine device.

Based on 2017 client data, most women adopting a voluntary PPIUD had either no education (61%) or primary education only (25%) (Figure 6). There were some differences in education status between public- and private-sector PPIUD clients; private-sector clients were less likely to have had no schooling and more likely to have primary and secondary education than clients accessing services in the public sector. The large proportion of women with no or little schooling accessing voluntary PPIUDs in both the public and private sector suggests that over the course of the dedicated PPIUD inserter pilot program service delivery successfully reached women of lower socioeconomic status, using education status as a proxy measure.

In 2017, 86% of women adopting a voluntary PPIUD had either no or only primary education.

**FIGURE 7:**
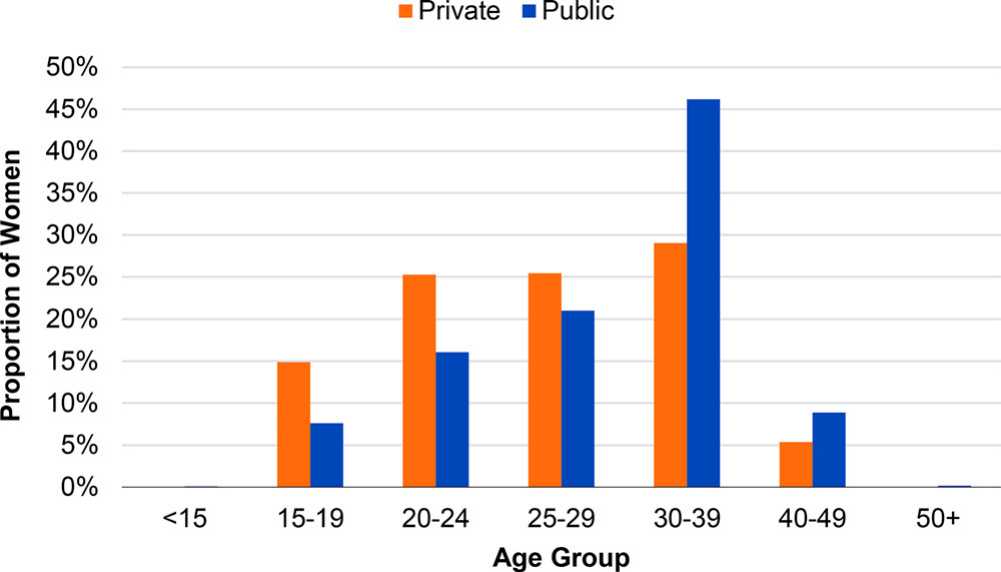
Age of Women Choosing a PPIUD, by Sector, 2017 Abbreviation: PPIUD, postpartum intrauterine device.

In 2017, although the proportion of women adopting a voluntary PPIUD was highest in the 30-to-39-year age group, over half (51%) of all women choosing a PPIUD were under the age of 30 (Figure 7). Almost half (46%) of clients in the public sector were aged 30 to 39 years compared to the private sector, where there was a much more even distribution across the age groups 20 to 24, 25 to 29, and 30 to 39 years.

The reasons for the differences in client age require further exploration. Based on interviews with some public providers, one explanation could be that multiparous women are more often identified as possible candidates for counseling for voluntary PPIUD in the public sector, while the private sector considers that all women of reproductive age may be interested in the PPIUD. Information regarding client parity was available in health-facility level registers but not available through internal management information system data and, therefore, was not included in this analysis.

## DISCUSSION

Since the introduction of the dedicated PPIUD inserter in Mali, the insertion technique has proved to be popular among service providers interviewed who consider it easier, faster, more convenient, and less risky than the conventional IUD inserted with forceps. Whether provider acceptability of the dedicated inserter has directly contributed to an increase in service results remains unclear due to multiple confounding factors identified during the pilot phase that may have influenced voluntary PPIUD uptake. Nevertheless, it can be considered that provider preference for and confidence with the inserter's use can contribute to reducing potential supply-side barriers. Historically, peaks in voluntary PPIUD services have coincided with training sessions for providers. This might suggest a potential downturn once trainings were reduced; however, since the program entered a more natural phase in August 2017, when trainings have reduced, the results have revealed stable or increasing numbers of monthly voluntary PPIUD services. While service trends in PPIUD service provision in the private facilities during the pilot phase steadily increased, they remained unpredictable for the public sector. They have, however, shown the potential of increasing access to PPIUD if providers are trained, confident, and motivated to provide the service. This was demonstrated during the training phases of providers; their motivation to qualify as a provider of the PPIUD with the dedicated inserter could be a contributing factor to the boost in figures. Despite the number of public and private providers trained on the dedicated PPIUD inserter, only a small proportion actively provide voluntary PPIUDs each month. This suggests that not all trained providers are operating at their full potential each month, perhaps due to factors such as a limited demand for PPIUD services or competing priorities other than PPFP counseling within maternity units.

Multiple confounding factors identified during the pilot phase may have influenced voluntary PPIUD uptake.

The large volume of deliveries in public-sector facilities in Mali demonstrates an opportunity to increase voluntary PPIUD access at scale, but a number of supportive factors—supplies, supportive supervision, and community sensitization—need to be in place to ensure uninterrupted delivery to all women, of all ages and parities. If providers in the public sector feel overstretched with their existing workload, consideration for how to ensure providers have the time, capacity, and motivation to integrate and sustain a new service is needed.

The large volume of deliveries in public-sector facilities shows an opportunity to increase voluntary PPIUD access at scale, but a number of supportive factors need to be in place to ensure uninterrupted delivery to all women.

While public facilities have a much greater number of deliveries, private facilities are providing voluntary PPIUDs to a much higher proportion of postpartum women, and more evenly across age groups. Further research is required to fully understand what is behind the differences between voluntary PPIUD uptake in public versus private facilities. A recent study in Nigeria identified strong uptake in private-sector facilities where 41% of women delivering chose a voluntary PPIUD.^21^ Once the determinants of strong uptake are better understood, there may be scope to apply learnings between sectors.

The dedicated PPIUD inserter may contribute to reducing some of the supply-side barriers that inhibit access to voluntary PPIUD services due to the inserter's acceptability among providers. However, this alone cannot increase uptake of postpartum contraception or of voluntary PPIUD as a proportion of the method mix. Integration of a new service into an already overstretched health system does not come without challenges. Further, Mali continues to face important demand-side barriers not only for PPIUD provision but also for the voluntary IUD and family planning in general, especially among men. Additional efforts are required to provide comprehensive information on birth spacing and family planning options at a community level. The importance of integration of PPFP services into different maternal health services was echoed by providers, with ANC identified as a critical time to counsel women on their PPFP options, especially for PPIUDs. Importance of PPFP counseling during all ANC sessions—for women of all ages and parity—should be stressed in future trainings and continue to be observed during onsite supervision visits. Understanding of the number of women who do not attend ANC sessions or return to facilities for postnatal care is equally important. PPFP strategies should include components to reach women in their homes or communities with PPFP information and referral pathways, or by integrating PPFP counseling into other maternal, infant, and child health services.^24^^,^^25^

PSI-Mali will continue to support the providers it has trained in the private and public sectors to provide quality, voluntary PPIUD services within the context of broad method choice to women in a critical stage of their reproductive life. Results from the dedicated inserter pilot program demonstrate the potential for this service to be scaled up, in order to enable women in Mali to achieve their fertility intentions and support their health and the health of their families.

### Limitations

Due to the limited scope of the case study, only a small number of providers could be interviewed. While they provided invaluable insights for PSI on the experiences of the dedicated PPIUD inserter and the drivers of voluntary PPIUD uptake, they were not considered to be a representative sample of PSI-trained providers, and therefore generalizable conclusions cannot be drawn. Additionally, the providers were all based in Bamako, the capital city; future research would benefit from including providers from other regions. Some of the calculations included in this case study are based on best-available information, such as the number of deliveries per facility, which are tallied manually in institutional log books and are, therefore, subject to potential human error.

## CONCLUSION

The dedicated PPIUD inserter has been widely accepted and preferred by providers in Mali and, where implemented, has potentially contributed to reducing some of the supply-side barriers associated with PPIUD services. While not directly attributable to the introduction of the dedicated inserter alone, voluntary PPIUD insertions have increased since its introduction, suggesting the potential for the dedicated PPIUD inserter to enhance access to PPIUD, given that only a small proportion of trained providers actively provide voluntary PPIUD insertions each month.

Further research is required to understand the impact of potential confounding factors to voluntary PPIUD uptake, the difference in trends between the public and private sectors, and client perspectives of the dedicated PPIUD inserter. However, the Mali pilot demonstrates notable acceptability and preference for the dedicated PPIUD inserter among a key constituent group who enable access to this important postpartum method, namely, providers. With continued support to providers, coupled with ongoing efforts to address demand-side barriers to PPIUD, and family planning more broadly, the dedicated PPIUD inserter could play an important role in responding to the high unmet need for postpartum women in Mali. On a global level, the lessons learned from Mali will inform the rollout of the dedicated PPIUD inserter taking place in 12 other countries. With increasing support for the scale up and integration of the dedicated PPIUD inserter in the pilot countries, and the WHO and United Nations Population Fund product prequalification processes underway, substantial potential exists for the dedicated PPIUD inserter to expand PPFP options, reduce unmet need for PPFP, and contribute to reducing maternal, infant, and child mortality.

By continuing to support providers and addressing demand-side barriers to PPIUD, the dedicated PPIUD inserter could play an important role in responding to the high unmet need for postpartum women in Mali.

## Notes

**En français**

**Aider les femmes post-partum au Mali à réaliser leurs intentions en matière de fertilité: perspectives de l'introduction du dispositif d'insertion dédié au DIU post-partum**

Dans cette contexte d'introduction pilote, les prestataires formés ont déclaré une acceptation et une préférence plus élevées pour le dispositif d'insertion dédié par rapport à l'insertion post-partum conventionnelle avec forceps, suggérant le potentiel du dispositif d'insertion dédié à élargir l'accès aux DIU post-partum

**RÉSUMÉ**

**Contexte :** Le Mali a l'un des taux de mortalité maternelle les plus élevés du monde, associé à l'un des taux les plus faibles d'utilisation de contraceptifs modernes. Près d'un quart des 750 000 naissances annuelles du pays surviennent dans les 24 mois suivants une naissance, ce qui augmente les risques pour les mères et les bébés. Près de 70 % des femmes en post-partum ont un besoin de planification familiale non satisfait. En 2016, Population Services International Mali (PSI-Mali) a introduit un applicateur spécialisé du dispositif intra-utérin du post-partum (DIUPP) pour remplacer la technique d'utilisation des forceps pour l'insertion du DIUPP ; ceci dans le but d'aider à résoudre ce déficit important de la planification familiale. Méthodes : Une approche à méthodes mixtes a été utilisée pour évaluer les résultats du programme et les expériences des prestataires formés par PSI qui utilisent l'applicateur spécialisé du DIUPP dans 5 établissements de santé à Bamako. Au total, 10 entretiens avec des informateurs clés ont été menés chez les prestataires, de même que 4 entretiens avec des informateurs clés du personnel opérationnel et clinique de PSI-Mali impliqué dans la formation et le soutien des prestataires. Des données supplémentaires ont été collectées à partir d'enquêtes sanitaires de district et de registres d'établissement. Les données secondaires englobaient la documentation de 2011 à 2017, avec les chiffres de prestation de services du DIUPP qui ont utilisé l'applicateur spécialisé sur la période pilote de mars 2016 à décembre 2017. Les données primaires ont été collectées au Mali en juillet 2017.

**Résultats :** Entre mars 2016 et décembre 2017, PSI-Mali a formé 134 prestataires du secteur public et privé sur l'applicateur spécialisé du DIUPP et fourni plus de 3 500 DIUPP volontaires. Sur les 1 840 DIUPP volontaires fournis en 2017 uniquement, 67 % ont été fournis par des établissements formés à l'utilisation de l'applicateur spécialisé du DIUPP. Les prestataires ont exprimé une préférence pour l'applicateur (par rapport à l'utilisation de forceps) en raison de sa facilité, de sa rapidité, et ont perçu les risques plus faibles d'infection associés. Les données de service des 5 établissements visités ont révélé une moyenne générale d'adoption du DIUPP de 7,3 % des accouchements en 2017. Bien que les établissements privés aient considérablement moins d'accouchements que les établissements publics (600 à 900 contre 20 à 30 respectivement), une proportion beaucoup plus élevée de femmes qui accouchent dans les établissements privés ont choisi un DIUPP. Les prestataires ont également évoqué d'autres obstacles du côté de l'offre et du côté de la demande continue qui empêchent l'adoption du DIUPP.

**Conclusion :** L'acceptation par les prestataires de l'applicateur spécialisé du DIUPP peut aider à réduire certains des obstacles du côté de l'offre qui empêchent les femmes d'accéder aux méthodes de planification familiale du post-partum. L'applicateur spécialisé du DIUPP pourrait jouer un rôle important en réponse au besoin élevé non satisfait de planification familiale chez les femmes en post-partum au Mali avec en synergie : un soutien continu aux prestataires ; des efforts continus pour résoudre les différences dans les tendances de service entre les secteurs et les obstacles du côté de la demande du DIUPP et de la planification familiale de manière plus générale.

## Notes

**Peer Reviewed**

**Funding**: This work was made possible by the support of the American people through the United States Agency for International Development (USAID) under the Support for International Family Planning Organizations 2 Project (Cooperative Agreement No. AID-OAA-A-14-00037) implemented by PSI.

**Disclaimer:** The contents are the responsibility of PSI and do not necessary reflect the views of USAID or the United States Government.

**First Published Online:** September 19, 2018
